# The effect of Tuina based on the concept of hip-knee-ankle conjugation in patients with chronic ankle instability: study protocol for a randomized controlled trial

**DOI:** 10.3389/fresc.2023.1165548

**Published:** 2023-08-25

**Authors:** Zonglin Wen, Ruoyun Lyu, Wei Wang, Xia Hua, Yifeng Yu, Ke Zeng, Lingjun Kong, JianWei Wang

**Affiliations:** ^1^ Department of Tuina, Shuguang Hospital Affiliated to Shanghai University of Traditional Chinese Medicine, Shanghai, China; ^2^ Department of Traditional Chinese Medicine, Shanghai Tenth People's Hospital of Tongji University, Shanghai, China

**Keywords:** chronic ankle instability, tuina, hip-knee-ankle conjugation, clinical trial, functional training, physical therapy

## Abstract

**Background:**

Chronic ankle instability (CAI) is a common sports injury disease and characterized by limited mobility, perceived instability and muscle weakness, combined treatment of hip-knee-ankle is a common rehabilitation method. Tuina, as a traditional Chinese manual therapy, is usually used for CAI, but many of them only focus on the local ankle joint rather than the combination of hip and knee joint. Therefore, we have designed a randomized controlled trial (RCT) to investigate the effects of Tuina base on the concept of hip-knee-ankle conjugation on the stability and balance of lower limbs and ankle function in patients with CAI.

**Methods:**

We have designed a randomized controlled trial. A total of 72 participants with CAI will be randomly divided into functional training groups and hip-knee-ankle Tuina combined with functional training group in a 1:1 ratio. Participants in control group will receive 8 sessions of functional training (30 min per session, twice a week for 4 weeks). Participants in intervention group will receive 8 sessions of Tuina combined with functional training (twice a week for 4 weeks). The primary outcomes include the Y-Balance Test (YBT) and Cumberland Ankle Instability Tool (CAIT). The Secondary outcomes include the Foot and Ankle Ability Measure (FAAM) and ankle range of motion (ROM). The outcome assessments will be conducted before the first intervention and after the last intervention.

**Discussion:**

The aim of this study is to explore a safe and effective manipulation program and serve as reference for clinical treatment of CAI and expect to provide the necessary theoretical and practical support to our future research.

**Clinical Trial Registration:**

Chinese Clinical Trail Registry ChiCTR2300068274.

## 1. Background

Ankle sprains are one of the most common recurrent injury disease in sports, according to statistics, ankle sprains account for 15% of all sports injuries (1), and over 40% of ankle sprains go on to develop chronic symptoms (2). Studies (3) have shown that the patients who suffer from ankle sprains are prone to ankle sprain again due to the lack of awareness and the failure of timely, effective treatment. Recurrent ankle sprains are one of the main causes of chronic ankle instability (CAI). CAI is associated with functional changes such as proprioception, neuromuscular control, muscle strength, and postural control. Another study shows that CAI (4) is deficient in strength and dynamic postural control of hip and knee extensors. Inappropriate joint biomechanics is the main risk factor of osteoarthritis (5). However, rehabilitation exercises enhancing the strength of the hip and knee muscles may have a positive effect on improving dynamic postural control.

The knee and ankle are adjacent joints. The flexion and extension of the two joints are closely related. The flexion and extension of the knee can reduce the unnecessary movement of ankle to protect the lower limb joints. Meanwhile, individuals with chronic ankle instability have insufficient ankle plantar flexion torque, which may result in insufficient knee flexor and extensor torque, as well as deficient neuromuscular adaptation of the knee (6). In addition, altering foot progression angle in both toe-in and toe-out directions significantly affects the external knee joint moments in patients with medial knee osteoarthritis (7). The external knee joint moments is a surrogate indicator of knee joint load distribution during walking (8), which is closely related to the progression of knee medial compartment osteoarthritis (9, 10). On the other hand, the hip joint is also an important joint to maintain the stability of human posture. Multiple studies (11–14) have shown that repetitive ankle injuries lead to decreased isometric strength of hip adductor and abductor muscles, while proximal neuromuscular injuries may exacerbate dynamic postural control deficits in people with ankle injuries and further affect central regulation of motor control, which ultimately lead to widespread lower limb neuromuscular injuries. Patients with ankle injury have delayed activation of the hip extensor during hip extension, which may be related to the afferent disturbance of the proximal limb muscles caused by the dysfunction of mechanoreceptor afferents in the distal injured ankle (15). Therefore, in the treatment of ankle injuries, the combined treatment of hip and knee joint should also be paid sufficient attention.

In 2021, the Academy of Orthopedics Physical Therapy of the American Physical Therapy Association (APTA) issued the clinical practice guide “Ankle stability and movement coordination impairments: lateral ankle ligament sprains revision 2021”. The guideline recommends manipulative therapy as one of the main treatments for ankle injuries. Tuina, as a traditional Chinese manual therapy, is often used to improve pain, range of motion, and tissue extensibility (16). According to traditional Chinese medicine, Tuina technique, such as Rotating-Traction-Poking manipulation and Rubbing-Traction-Kneading manipulation and so on, can activate blood and remove stasis, relieve swelling and pain (17), and achieve the purpose of releasing soft tissue spasm, restoring joint alignment, improving ankle function and promoting rehabilitation (18–21). There are also some evidence to suggest that manual therapy can improve ankle dorsiflexion range of motion or dynamic postural control in patients with CAI (22, 23). However, in the treatment of CAI, many clinicians only focus on the local ankle joint rather than the combination of hip and knee joint.

Traditional Chinese medicine is highly affected by the concept of viewing things of Chinese classical philosophy, the concept of holism permeates through all the fields in traditional Chinese medicine, including physiology, pathology, diagnosis, syndrome differentiation and treatment. Traditional Chinese medicine believes that the human body is an organic whole, including hip-knee-ankle. In the treatment of CAI, it suggests that we should focus overall rather than only focus on the local to achieve better therapeutic effect. As an important part of traditional Chinese medicine, the concept of holism is also the principle of Tuina therapy.

The guideline also suggests that functional training is an important part of the treatment of ankle injuries (24), such as proprioceptive training, balance training and muscle strength training and so on. Many studies (25–28) have shown that functional training can enhance ankle stability, improve balance ability, reduce the risk of re-injury, promote the rehabilitation of ankle injury and help patients return to normal life and work as soon as possible.

Therefore, based on the concept of hip-knee-ankle conjugation, the objective of this randomized controlled trial (RCT) is to investigate the effects of 4-weeks Tuina on stability and balance of lower limbs, ankle function and ankle range of motion (ROM) of CAI patients to explore a new manipulation program for the treatment of ankle injuries.

## 2. Methods/design

### 2.1. Objectives

To investigate the effects of Tuina on stability and balance of lower limbs, ankle function and ankle range of motion (ROM) on CAI patients and explore a new manipulation program for the treatment of ankle injuries.

### 2.2. Trial design

This study is a randomized controlled trial (RCT) designed to confirm the clinical efficacy of Tuina therapy base on the concept of hip-knee-ankle conjugation in CAI. Seventy-two participants with CAI will be randomly assigned into intervention group and control group with the ratio of 1:1.

### 2.3. Participants

#### 2.3.1. Inclusion criteria

(1) According to the position statement of the International Ankle Consortium criteria (29).
(2) Participants have a history of at least one severe ankle sprain in the 12 months prior to join in this study, with pain, swelling and other symptoms on the affected side, which resulted in at least one day of inability to function normally.
(3) Two or more episodes of ankle loss of control and/or sprain and/or instability within 6 months.
(4) Cumberland Ankle Instability Tool (CAIT) scores less or equal than 27.
(5) Age 18–60 years old, male or female.

#### 2.3.2. Exclusion criteria

(1) Cardiovascular, hepatic, renal, hematopoietic, and other serious primary diseases or mental disorders.
(2) Pregnant or lactating women who cannot complete the radiology examination.
(3) Patients with rheumatism/rheumatoid arthritis, osteoporosis, gout and other serious bone and joint diseases.
(4) Those patients who could not correctly understand and fill in various scales related to the study.

#### 2.3.3. Suspension criteria

(1) The condition deteriorates, or more serious complications occur.
(2) Serious adverse reactions occurred.

#### 2.3.4. Dropout criteria

(1) Participants do not follow the original treatment plan (poor compliance).
(2) Participants receive other treatments without investigator's permission during the intervention period.
(3) Participants withdrew from the trial due to their own factors.

#### 2.3.5. Recruitment

Patients with CAI will be recruited from Tuina Department of Shuguang Hospital affiliated to Shanghai University of Traditional Chinese Medicine. The main recruitment method will be advertising on social media, such as WeChat and Weibo, which is like WhatsApp and Twitter. The poster will also be displayed in hospital.

#### 2.3.6. Patient safety

The intensity of Tuina treatment should not be too large and the duration of treatment should not be excessive. Participants will be reminded to be careful during the test. Participants with aggravation of pain or other serious complications after the Tuina treatment will be referred to an orthopedist or surgeon to receive other form of treatment.

### 2.4. Intervention

We will explain the details of our study to the participants before they join our study. The frequency of treatment is twice a week for 4 weeks.

#### 2.4.1. Functional training group (control group)

In this group, we will divide the functional training into two parts.

(1) Balance training

Squat with eyes closed: participants will squat and stand up slowly with eyes closed. At the beginning, they can support the wall or help objects, gradually increase the amplitude and reduce dependence. During each session, they need to perform three sets of ten repetitions.

Balance board training: participants will stand on the balance board with both feet, maintain balance and slightly rock from side to side. At the beginning, they can support the wall or help objects, and the difficulty can gradually increase. During each session, they need to perform three sets of ten repetitions.

(2) Resistance training

During each session, participants will use the resistance band to complete the resistance training in four directions of ankle motion (plantar flexion, dorsiflexion, inversion and eversion) which perform 3 sets of 10 repetitions.

#### 2.4.2. Hip-knee-ankle tuina and functional training group (intervention group)

In this group, the treatment scheme is based on the control group, and we will add the Tuina therapy base on the concept of hip-knee-ankle conjugation which is shown below. We will establish a scientific and normative, standardization work process, execute operation procedures and quality standards seriously. All treatment will be performed by the registered Tuina doctors, who have the qualification of clinicians and have been engaged in clinical work of tuina for more than 5 years. All of them graduated from the major of Tuina, and they will be trained and assessed with the standard operating procedures (SOP) of tuina manipulation in this study. Each session of Tuina treatment lasts about 30 min.

First, participants will lie in a supine position, with legs straight and relax. The doctor will hold the ankle with both hands, place the thumb on the patient's lateral or medial malleolus suture and press, while the other four fingers fix, rotate and shake the foot for several times. The ankle is pulled outward and downward with both thumbs. Then, keep kneading and twirling on the ankle relax tendons and activate collaterals and promote recovery. Finally, rub the ankle and taken heat transmission as the degree.

Second, participants keep lying in a supine position, the doctor will relax the soft tissues of the groin, thigh, knee and lower leg in turn, including quadriceps femoris, tibialis anterior muscle and soft tissues around the patella and so on. According to the actual situation, the knee adjustment manipulation techniques in sitting or supine position will be performed when necessary.

Third, participants transform in a prone position, the doctor will relax the muscles of the buttocks, thighs and calves in turn, including gluteus maximus, gluteus medius, piriformis, biceps femoris and gastrocnemius and so on. The manipulation should not be too heavy. Take the feeling of acid, swelling and numbness as degree. According to the actual situation, the hip joint adjustment manipulation techniques will be performed when necessary.

### 2.5. Outcome measures

#### 2.5.1. Primary outcome measures

##### 2.5.1.1. Y-balance test (YBT)

The first primary outcome measure is the stability and balance of lower limbs evaluated by the Y-Balance Test (YBT). Y-Balance Test is a clinical instrument base on star excursion balance test (SEBT) which is commonly used for assesses dynamic stability and neuromuscular control of the lower extremity, and studies have proofed its reliability (30). The YBT will be assessed before and after Tuina treatment.

Before the YBT, participants will be instructed to learn the correct stretch technique and allowed four practice trials in each direction, with three consecutive test trials in each direction during testing. The order of the directions is random. Composite scores and two-sided differences will be calculated at the end of the test. The calculation formula is shown below. If the composite score is less than 95%, it indicates poor joint stability, and there may be a high risk of injury to the support leg, a lower score indicates a higher risk of injury. If bilateral difference is greater than 5%, it suggests a large difference in strength or balance between the left and right support legs, a higher difference indicates a higher risk. Studies have shown that patients with an anterior right/left reach distance difference greater than 4 cm would be 2.5 times more likely to sustain a lower extremity injury (31).

$$\mathrm{Composite}\;\mathrm{score}=\frac{a+b+c}{L\times 3}\times 100\text{\%}$$

$$\mathrm{Two}\text{-}\mathrm{sided}\;\mathrm{differences}=\frac{\left(a1+b1+c1)-(a2+b2+c2\right)}{(a1+b1+c1+a2+b2+c2)/2}\times 100\text{\%}$$

Notes: *a*1, *b*1, *c*1, *a*2, *b*2 and *c*2 represent the farthest distance of the left/right foot extended in the three directions respectively. *L* represents the length of the leg.

##### 2.5.1.2. Cumberland ankle instability tool (CAIT)

The second primary outcome measure is self-assessment of ankle instability evaluated by Cumberland Ankle Instability Tool (CAIT). CAIT is an easy and understandable valid tool for the assessment of chronic ankle instability (32), and it can differentiate the stable and unstable ankle (33). Participants will rate their left and right ankles separately. Each question is assigned a different score based on the number of options. The maximum unilateral ankle score is 30. Participants with a unilateral ankle score equal or less than 27 are considered unstable on that side of the ankle and the range above it are considered stable (33).

#### 2.5.2. Secondary outcome measures

##### 2.5.2.1. Ankle function

Ankle function will be assessed by Foot and Ankle Ability Measure (FAAM). The FAAM is also a patient-completed rating scale consisting of a daily activities subscale (21 rated items) and a physical activity subscale (7 rated items) with response options on a 5-point Likert scale (4 to 0, respectively), which has been demonstrated reliable, to evaluate postural control and muscle strength in patients with CAI (34).

##### 2.5.2.2. Ankle range of motion

Ankle range of motion (ROM) will be assessed by limb goniometer. Ankle plantar flexion/dorsiflexion, varus and valgus range of motion will be measured, the method of measurement is shown below, and the angle of activity will be measured and recorded by the researcher.

(1) Ankle dorsiflexion range of motion: participants will move their weight forward, squat on one leg, land on their heels, and flex their knees to the maximum. The angle between the lower leg and the ground will be measured when the ankle is dorsiflexed to the maximum range of motion.
(2) Ankle plantarflexion range of motion: participants will move their weight back, squat on one leg, land on the balls of their feet, and extend their knees to the maximum extent. The angle between the lower leg and the ground will be measured when the ankle is plantarflexed to the maximum range of motion.
(3) Range of motion of ankle inversion and eversion: participants will move their weight to the left and right sides, squat on one leg, land on the balls of their feet, and maximize inversion and eversion their ankle. The angle between the lower leg and the ground will be measured when the ankle inversion and eversion to the maximum range of motion.

### 2.6. Sample size calculation

The participants will be randomly divided into functional training group (control group) and hip-knee-ankle Tuina group (intervention group), the allocation ratio of the two groups is 1:1. Sample size calculation is based on the Y-Balance Test (the primary outcome), the sample size formula for comparison of means between two groups in a randomized controlled trial is used to perform the sample size calculation. With the test levels set at *α* = 0.05 (2-sided), *β* = 0.1, according to the relevant literature at home and abroad (31, 35), the maximum acceptable right/left reach distance difference was 4 cm. The standard deviation of the comprehensive score of Y-Balance test in the pre-experimental observation was 4.95. It is substituted into the sample size calculation formula, *n* = 32.15. Considering a 10% dropout rate, and take the integer *n* = 36, so we will include 36 participants for each group, the total number of participants is 72.

### 2.7. Randomization, allocation, and blinding

We will use Statistical Package for the Social Sciences (SPSS) Ver.25.0 (IBM Inc, New York, USA) and manual assistance to complete randomization, the method is shown below.

Before the study, a randomization specialist, who will not join other part of this trial, will be set up, and the specialist will use SPSS Ver.25.0 to generate a set of random numbers according to the numbers 1–72. After that, the numbers will be sorted according to the order of random number from small to large, 72 numbers will be randomly divided into the intervention group and the control group, and the numbers and corresponding groups will be sent to the researchers. When the appropriate participants are recruited, the researchers will enter the form and number it according to the order of enrollment, and determined the group of the current case according to the grouping generated by the randomization specialist. The lists of the two groups are sorted into tables and sent to the doctor, who are informed of the treatment plan for each group in advance, in order to reduce unnecessary communication between researchers and randomization specialist in the grouping process, and avoid the influence of researchers' subjective will on the grouping as much as possible. The participants and the doctor will not be blinded because of specialization of Tuina. However, the data analysis and efficacy assessment will be performed by the research assistant, who will not join other part of this trial, and blinded in order to decrease analysis bias.

### 2.8. Data collection and management

We will collect the baseline data before the study. The primary and secondary outcomes will be assessed at baseline and 4 weeks (at the end of the intervention). The researcher will collect the data in the case report form (CRF) after obtaining signed consent from the participants. To protect the privacy of the participants, we will use codes and initials instead of their personal information. After data uploading and confirming, only statistician can access to the data.

### 2.9. Statistical analysis

The principle of Intention-To-Treat (ITT) analysis and Per-Protocol (PP) analysis will be followed in the process of statistical analysis. The Last Observation Carried Forward Analysis (LOCF) rule will be applied for missing data in ITT analysis. All the statistical analysis will be performed using SPSS Ver.25.0 with a significance level of 0.05.

First, the different baseline characteristics of the two groups will be described, data will be presented as mean or median with standard deviation or interquartile range for continuous variables and as frequency distributions for categorical variables. Moreover, the histograms, boxes and other representations will also be considered for the graphic analysis of corresponding data. Two-sample *t*-test for quantitative data or Chi-square test for qualitative data will be performed as homogeneity test, and the covariance analysis will also be performed if an adjustment is needed for a baseline characteristic.

Second, the analyses will focus on whether statistically better treatment outcomes could be achieved in the intervention group. *p* Values less than 0.05 were considered statistically significant and tests were 2-sided.

Third, safety analysis will be performed using the Chi-square test or Fisher's exact test based on the frequency and percentage of all recorded adverse events between two groups.

### 2.10. Flow chart

The Flow chart is detailed in Appendix I below.

## 3. Discussion

Holism is one of the characteristics of traditional Chinese medicine, during the process of diagnosing disease and treatment, how to apply the holistic concept and syndrome differentiation is one of the most important problems in clinical treatment. In the treatment of CAI, Tuina therapy is one of the common and effective treatment methods, and it can also combine the treatment of hip-knee-ankle in a good way, so we have designed a clinical trial for the application of Tuina in the treatment of chronic ankle instability. The aim of this study is to investigate the effects of Tuina based on the concept of hip-knee-ankle conjugation in patients with chronic ankle instability. The stability and balance of lower limbs will be assessed by Y-Balance Test and CAIT scores. The ankle function will be assessed by FAAM scores and ankle range of motion test. The study will attempt to explore the effect of hip-knee-ankle treatment on chronic ankle instability and provide evidence of the effects of Tuina in patients with CAI.

Decreased balance and impaired precise joint movement control are the main consequences of CAI, and recurrent ankle sprain is one of the important causes of this disease. These symptoms ultimately degrade the quality of life and athletic ability of patients with CAI. Although there are previous studies on hip-knee-ankle conjugate theory, they are most applied in the treatment of knee osteoarthritis. This study focus on the effect of hip-knee-ankle conjugate theory on the treatment of CAI, in order to explore how to improve the stability and balance of lower limbs and ankle function by using manipulation and improve the quality of life and motor ability of the patients with CAI.

Although the aim of this trial is to explore the effect of Tuina on ankle injuries and address the limitations of previous studies, this study also has its own limitations. First, because it is impossible to blind the doctor and participants, it is difficult to design a double-blinded RCT. To minimize the risk of detection bias, we have a randomization specialist, he will divide the participants into two groups randomly prior to the study, the assessors and analyst will be blinded to the group assignment in this study. Second, due to lack of precision instruments, lower limb stability, balance ability and ankle range of motion can only be assessed manually, which inevitably leads to certain errors.

In summary, we have designed a randomized controlled clinical trial to explore a safe and effective manipulation program to serve as reference for clinical treatment of ankle injuries. We also expect that this study can help our future research to provide the necessary theoretical and practical support.

## 4. Trial status

The version number of this protocol is 2.0, dated on 10 January 2023. Participants will be recruited between March 2023 and June 2024. Study completion is expected to be October 2024.

## Ethics statement

The studies involving human participants were reviewed and approved by IRB of Shuguang Hospital afiliated with Shanghai University of Traditional Chinese Medicine (Approved number of ethic committee: 2022-1226-163-01). The patients/participants provided their written informed consent to participate in this study.

## Figures and Tables

**Appendix Figure 1:**
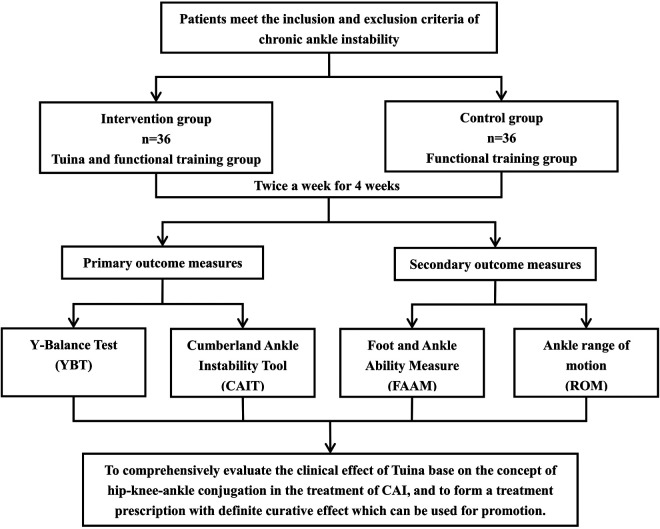
Flow Chart
